# Is there a link between the extracranial venous system and central nervous system pathology?

**DOI:** 10.1186/1741-7015-11-259

**Published:** 2013-12-17

**Authors:** Robert Zivadinov

**Affiliations:** 1 Buffalo Neuroimaging Analysis Center, Department of Neurology, School of Medicine and Biomedical Sciences, University at Buffalo, State University of New York, Buffalo, NY, USA; 2 MRI Clinical Translational Research Center, School of Medicine and Biomedical Sciences, University at Buffalo, State University of New York, Buffalo, NY, USA

**Keywords:** Extracranial venous system, Jugular venous reflux, Chronic cerebrospinal venous insufficiency, CNS pathology, Aging

## Abstract

The extracranial venous system is complex and variable between individuals. Until recently, these variations were acknowledged as developmental variants and were not considered pathological findings. However, in the last decade, the presence and severity of uni- or bi-lateral jugular venous reflux (JVR) was linked to several central nervous system (CNS) disorders such as transient global amnesia, transient monocular blindness, cough headache, primary exertional headache and, most recently, to Alzheimer's disease. The most recent introduction of a composite criteria-based vascular condition named chronic cerebrospinal venous insufficiency (CCSVI), which was originally linked to multiple sclerosis, increased the interest in better understanding the role of the extracranial venous system in the pathophysiology of CNS disorders. The ultimate cause-consequence relationship between these conditions and CNS disorders has not been firmly established and further research is needed. The purpose of this article collection in *BMC Medicine* and *BMC Neurology* is to synthesize current concepts and most recent findings concerning the evaluation, etiology, pathophysiology and clinical relevance of the potential involvement of the extracranial venous system in the pathology of multiple CNS disorders and in aging.

Please see related debate: http://www.biomedcentral.com/1741-7015/11/260.

## Editorial

The potential involvement of the extracranial venous system in the pathology of central nervous system (CNS) disorders and in aging is largely debated at this time [1,2]. This is primarily because the role of venous drainage impairment, for example, extracranial venous abnormalities, is largely unknown and because the venous system is subject to many variations [1-4]. However, the knowledge has been rapidly changing with regards to the role of the extracranial venous system in relation to CNS pathology. This is because a range of extracranial venous abnormalities that disrupt normal blood flow and are characterized with the development of prominent collateral circulation, have recently been associated with a number of CNS disorders and aging [3,5-8].

The extracranial venous system is complex with variability between individuals and is frequently asymmetric [3]. Until recently, these variations were acknowledged as developmental variants and were not considered pathological findings [9]. In the last decade, the presence and severity of uni- or bi-lateral jugular venous reflux (JVR) was linked to several CNS disorders, such as transient global amnesia, transient monocular blindness, cough headache, primary exertional headache and, most recently, to Alzheimer's disease [8,10-16]. However, the most recent introduction of a composite criteria-based vascular condition named chronic cerebrospinal venous insufficiency (CCSVI) [6] increased the interest in better understanding the role of the extracranial venous system in the pathophysiology of CNS disorders [5]. CCSVI is characterized by extracranial cerebrospinal venous outflow abnormalities of the main routes that may interfere with normal venous outflow. These anomalies were described in the internal jugular veins (IJVs), the vertebral veins and the azygos vein.

The debate article by Zivadinov and Chung [5] provides a timely update about the anatomy, etiology and pathophysiology of the extracranial venous system abnormalities/developmental variants and their potential involvement with CNS pathology. A classification of extracranial venous abnormalities/developmental variants that is independent of any single diagnostic imaging modality is presented. These can be classified as structural/morphological, hemodynamic/functional and those determined only by the composite criteria and use of multimodal imaging. Furthermore, structural/morphological venous abnormalities are divided into those creating narrowing or occlusion and those causing abnormal distensibility, whereas hemodynamic functional venous abnormalities are classified as abnormal cerebral venous outflow in the presence or absence of a structural venous anomaly in the extracranial veins. Zivadinov and Chung point out that one of the central issues to be further investigated is the definition of the significant narrowing of the extracranial venous system with hemodynamic consequences for the intracranial venous drainage. They critically report that the current definition (narrowing of >50% in respect to the proximal adjacent vein segment) is mainly derived from observations in the arterial system and is, therefore, probably inadequate for the venous system. The article also examines the association of these venous abnormalities, as well as their clinical correlates in relation to various CNS disorders and aging. Two other original articles of this article collection examine the relationship between the presence and severity of CCSVI and cognitive dysfunction in patients with multiple sclerosis (MS) [17,18]. Benedict *et al.* examined 109 MS patients and found no evidence of an association between the presence and severity of CCSVI with cognitive impairment and depression in patients with MS [17], while Leone *et al.* evaluated 61 MS patients and found no association between CCSVI and cognitive impairment, fatigue, depression, bladder/sexual symptoms and self-reported quality of life [18]. These findings are corroborating numerous other recent clinical and magnetic resonance imaging (MRI) studies that showed no evidence of a clinical association between CCSVI and MS [19,20].

The limited understanding of the pathophysiology of the extracranial venous system may consequently underestimate the impact of cerebral venous drainage abnormalities in a variety of CNS disorders [5]. Because of this, there is a need for more basic science and clinical studies to increase our knowledge and understanding of the clinical association and pathophysiologies of cerebral venous drainage abnormalities. In the review article by CB Beggs [21], the author discusses the pathophysiology regarding venous abnormalities in MS, leukoaraiosis and normal-pressure hydrocephalus (NPH). The review is supplemented with a hydrodynamic analysis to assess the effects on cerebrospinal fluid (CSF) dynamics and cerebral blood flow (CBF) of venous hypertension in general and CCSVI in particular. An obstruction of the extracranial venous drainage pathways may result in hypoxia. It is underlined that the hydrodynamic properties of the periventricular veins make these vessels particularly vulnerable to ischemia and plaque formation which may explain their frequent involvement in MS, leukoaraiosis and other neurodegenerative CNS diseases. The review also provides evidence that venous hypertension in the dural sinuses can alter intracranial compliance and change the CSF dynamics which is observed both in patients with MS and NPH.

One of the most convincing indicators towards a “vascular origin” for MS comes from neuropathological observations showing that MS plaques are exclusively perivenular and dimensions of the veins determine the shape, course and dimension of the lesions [22]. This is supported by recent imaging studies showing that a majority of MS lesions are associated with centrally coursing veins [23]. Such findings raise fundamental questions about the nature of this disease, that is, why their pathognomonic lesions do not develop around the arteries and what exactly are the roles of cerebral venous inflammation in their pathogenesis. A review article by Alexander *et al.*[24] presents a comprehensive review of the pathophysiology of MS, acute disseminated encephalomyelitis, pseudotumor cerebri and optic neuritis, with an emphasis on the roles of venous vascular system programming and dysfunction in their pathogenesis. They consider the fundamental differences between arterial and venous endothelium their dissimilar responses to inflammation and the potential theoretical contributions of chronic venous insufficiency in the pathogenesis of neurovascular diseases.

The pathophysiology of extracranial venous abnormalities may be further elucidated by exploring the role of precipitating risk factors [2,5,25,26]. The incidence and prevalence of extracranial venous abnormalities should be determined in relation to embryologic/developmental arrest factors, demographic factors (such as age, sex, race), cardiovascular risk factors (smoking, obesity, hypertension, diabetes, hyperlipidemia), inflammatory comorbidities and other possible precipitating risk factors, such as one’s level of exercise and diet [5]. In an original article, Chung *et al.*[27] tested the hypothesis that JVR, in addition to an increased level of plasma endothelin-1 (ET-1), a potent vasoconstrictor, is involved in the pathophysiology of cough syncope in 17 cough syncope or pre-syncope patients and in 51 age- and sex-matched healthy controls. The authors showed a synergistic effect between JVR and plasma ET-1 levels on the occurrence of cough syncope/pre-syncope. This is indeed an interesting finding, as it suggests that extracranial venous abnormality *per se* is not sufficient to increase the hydraulic resistance of the cerebral vascular bed but that other mechanisms must be at work, including the potential role of various precipitating risk factors.

At this time, there is no established diagnostic imaging modality, non-invasive or invasive, that can serve as the “gold standard” for the detection of any extracranial venous abnormalities [3]. However, consensus guidelines and standardized imaging protocols are emerging. Dolic *et al.* provide a comprehensive review of non-invasive and invasive imaging methods for the detection of extracranial venous abnormalities, including CCSVI [3]. They describe in detail the advantages and disadvantages of non-invasive imaging modalities such as Doppler sonography, magnetic resonance venography, computed tomography venography and plethysmography, as well as invasive imaging methods, including catheter venography and intravascular sonography. The article emphasizes the need for the use of composite criteria by uni- or multi-modal imaging modalities of the extracranial venous system because it is almost impossible to determine the relevance of a single structural/morphologic or hemodynamic/functional venous abnormality, regardless of the imaging modality or methodology utilized. In fact, in an original article, Zivadinov *et al.* evaluated the non-invasive and invasive multimodal imaging correlates of 20 MS patients with relapsing MS who were enrolled in the Prospective Randomized Endovascular therapy in Multiple Sclerosis” (PREMiSe) study [28]. They conclude that both a non-invasive and invasive multimodal imaging diagnostic approach should be recommended to depict a range of extracranial venous anomalies indicative of CCSVI.

When there is narrowing of the principal pathways of the extracranial venous system, collateral veins usually form as physiological secondary compensation for the compromised venous system outflow [3,4]. The presence of collateral flows, from a biomechanical point of view, is the strongest evidence for constricted principal venous pathways and venous hypertension. In an original pilot study, Zamboni *et al.* present a novel model in a clinical setting that suggests the pivotal role of the collateral network in draining the blood into the superior vena cava under CCSVI conditions [29]. One of the fundamental issues to be further investigated when determining the impact of significant extracranial venous narrowing is the degree of collateral circulation compensation.

While a link between the presence and severity of extracranial venous abnormalities and several CNS disorders as well as aging is emerging, it is to be determined whether those may play a potential role, as precipitating factors, to increased susceptibility for a number of CNS disorders. Although the presence and severity of JVR and CCSVI have been linked to a number of CNS disorders, the ultimate cause-consequence relationship has not been firmly established. In an original article, Cheng *et al.* showed in 23 transient monocular blindness (TMB) patients who had no carotid stenosis and in 23 age- and sex-matched healthy controls that there was a greater severity of IJV compression/stenosis in TMB patients [11]. Therefore, there is a future need for a better understanding of the role of extracranial venous abnormalities but many questions remain unanswered at this time. Because of this, the endovascular treatment for the correction of these extracranial venous abnormalities should be discouraged at this time, until the potential benefit is demonstrated in properly-designed blinded, randomized and controlled clinical trials.

The **‘**Venous Involvement in Neurological Disorders and Aging’ article collection in *BMC Medicine* and *BMC Neurology* has attempted to present a fair and balanced discussion of the examined topics. It is hoped that the contents of this collection will encourage the readers to continue their research on this subject.

## Abbreviations

CBF: Cerebral blood flow; CCSVI: Chronic cerebrospinal venous insufficiency; CNS: Central nervous system; CSF: Cerebrospinal fluid; ET-1: Endothelin-1; IJV: Internal jugular vein; JVR: Jugular venous reflux; MS: Multiple sclerosis; NPH: Normal-pressure hydrocephalus; PREMiSe: Prospective Randomized Endovascular therapy in Multiple Sclerosis; TMB: Transient monocular blindness.

## Competing interests

Robert Zivadinov received personal compensation from Teva Neuroscience, Biogen Idec, EMD Serono, Bayer, Genzyme-Sanofi, Novartis, Claret and General Electric for speaking and consultant fees. He received financial support for research activities from Biogen Idec, Teva Neuroscience, Genzyme-Sanofi, Novartis and EMD Serono.

## Authors’ information

Robert Zivadinov, M.D., Ph.D., is a professor of neurology with tenure at the Department of Neurology, School of Medicine and Biomedical Sciences, University of Buffalo, State University of New York (SUNY) and clinical professor of neurology at the Florida International University College of Medicine. He is director of the Buffalo Neuroimaging Analysis Center and of the MR Imaging Clinical Translational Research Center at the University of Buffalo.

He has performed extensive research in multiple sclerosis and imaging, having published more than 250 articles and 400 abstracts in leading peer-reviewed journals. He is currently pursuing research studies on quantitative magnetic resonance, ultrasound, angiography and optic coherence tomography imaging findings in multiple sclerosis, Parkinson’s and Alzheimer's disease and aging. His current interests also concentrate on therapeutic interventions, including strategies towards assessing neuroprotective efforts in neurodegenerative disorders, as well as venous function, genetic and neuroepidemiology fields of these diseases.

Dr. Zivadinov guest-edited this article collection in *BMC Medicine* and *BMC Neurology* with the aim of synthesizing most recent concepts concerning the evaluation, etiology, pathophysiology and clinical relevance of the potential involvement of the extracranial venous system in the pathology of multiple CNS disorders and in aging. Dr. Zivadinov is one of the leading authorities in the field on this topic, having published more than 30 original and review articles in peer-reviewed journals.

## Note

All articles in this article collection have been independently prepared by the authors and have been subject to the standard peer-review processes of the journals.
